# Making therapies culturally relevant: translation, cultural adaptation and field-testing of the Thinking Healthy Programme for perinatal depression in China

**DOI:** 10.1186/s12884-020-03044-1

**Published:** 2020-06-22

**Authors:** Anum Nisar, Juan Yin, Nan Yiping, Huo Lanting, Jingjun Zhang, Duolao Wang, Atif Rahman, Xiaomei Li

**Affiliations:** 1 Xi’an Jiaotong University, Xian, PR China; 2 Liverpool School of Tropical Medicine, Liverpool, UK; 3 University of Liverpool, Liverpool, UK

**Keywords:** Cultural adaptation, Field-testing, Thinking healthy Programme, Perinatal mental health, Maternal and child health

## Abstract

**Background:**

The prevalence of perinatal depression in China ranges from 15 to 20% and the vast majority of prenatally depressed women do not receive the intervention they require. Recent research evidence shows that evidence based, culturally–adapted psychosocial interventions are effective in reducing mental health problems. The World Health Organization (WHO) has endorsed the Thinking Healthy Programme (THP), which is an evidence based psychosocial intervention that can be delivered by non-mental health specialists. The aim of this study was to translate and adapt THP for the Chinese population and to establish its acceptability when delivered by non-specialists to a group of mothers with perinatal depression.

**Methods:**

The study was conducted in two phases. The THP manual, handbook, and health calendar was translated and adapted based on 8 domains of the Bernal framework (language, metaphors, content, concepts, goals, context, people and methods). Pre-testing was done using cognitive interviewing in the first phase. In second phase of field-testing, THP sessions were delivered to the depressed women by local THP trained nurses. Post intervention, programme survey was used for evaluation.

**Results:**

This study showed that the core structure, process and techniques of the THP were culturally compatible with the target Chinese population and did not require major changes. It was found that the adapted version of THP manual, handbook, and health calendar were acceptable, understandable, and culturally relevant to the Chinese women and their family members. Nurses were found as a suitable delivery agent by the mothers and their families.

**Conclusion:**

The Thinking Healthy Programme is acceptable and transferable to the Chinese cultural and healthcare context and nurses are a suitable delivery agent. The translated and adapted version of THP can be used for further implementation and evaluation studies in the Chinese context. Further evaluation can help establish the effectiveness of the programme and barriers to scale-up in China.

## Background

Maternal depression is the second leading cause of disease burden in women worldwide [1]. An episode of depression experienced during perinatal period, which is generally considered from pregnancy to one year after delivery, is classified as perinatal depression [2, 3]. The prevalence of perinatal depression, which includes prenatal as well as postnatal depression, is 10–15% in high-income countries and 15–20% in low and middle-income countries (LMICs) [4–6]. Perinatal depression is associated with increased functional impairment, reduced self-care, reduced social support and poor nutrition as well as increased risk of preeclampsia, and other pregnancy and labor complications in the women [7–9]. In children, the consequences of perinatal depression can lead to conditions like preterm and low weight birth, poor immunization rates, high rates of diarrhea and other infectious diseases, poor cognitive development and emotional disorders, disruptive behaviors, and poor academic performance [10–13]. Thus, perinatal depression is fast assuming significance as a global public health priority [1].

As the world’s most populous country, China accounts for about a fifth of the world’s population. The prevalence of perinatal depression ranges from 15 to 20% and according to recent pregnancy and childbirth estimates it translates to potentially 5 to 7 million women with perinatal depression [14, 15]. In contrast, there are only 1.7 psychiatrists and 0.05 mental health hospitals per 100,000 people [16]. Besides the shortage of health care human resources, equity of health-care is a big challenge. The total number of health institutions is same in Eastern and Western regions of China, but the health care workforce is much higher in the Eastern developed regions (3·7 million) compared to Western less-developed ones (2·2 million) [17]. However, the prevalence of perinatal depression is higher in the under-developed regions compared with that of the developed ones, based on a recent systematic review of 96 studies from 23 regions of China [Unpublished observation, Anum Nisar, Juan Yin, Ahmed Waqas. Prevalence of perinatal depression and its determinants in Mainland China: A systematic review and meta-analysis. 2019]. Thus, those in greatest need for service are least likely to have access to it, and this is especially true for mental health conditions [16].

Psychological therapies, including cognitive behavioural, interpersonal, supportive and group therapy, are recommended as the front-line management for perinatal depression [18]. In High-Income countries, such interventions are generally provided by mental health specialists, while in LMICs mental health service provision is low due to scarcity of specialized mental health resources. A systematic review of interventions for pre- and postnatal depression in low- and middle-income countries has found that evidence-based interventions delivered by non-specialists can be effective in treating the condition, as well as improving outcomes in the children of treated mothers [19]. Interventions include cognitive behaviour therapy and interpersonal therapy, as well as supportive and psycho- educational interventions. Despite this evidence, the vast majority of women with the condition remain untreated. To bridge the treatment gap, The World Health Organization (WHO), based on this review of available evidence for such interventions in LMICs, recommended the Thinking Healthy Programme (THP) as a therapy of choice for women requiring psychological intervention for perinatal depression in primary health care settings [20]. The Thinking Healthy Programme was originally developed in Pakistan after thorough formative research and robust evaluation through a randomized controlled trial [21, 22]. It employs specific as well as non-specific elements of Cognitive Behaviour Therapy (CBT), such as building an empathetic relationship, focusing on the here and now, behaviour activation and problem solving. The programme is fully manualized and has culturally appropriate pictorial illustrations aimed at helping mothers reflect on their thinking process and encouraging family support. The sessions are organised into five modules covering the period from the third trimester of pregnancy to one-year postnatal. THP, delivered by community health workers, was tested in a large community-based randomized controlled trial in Pakistan, where it more than halved the prevalence of perinatal depression and significantly improved child health outcomes like diarrheal episodes and vaccination coverage [22]. A key feature of the THP is that non-specialists can be trained to deliver the programme under specialist’s supervision, and the intervention can be integrated into primary health care system. Thus, it is ideally suited to regions where there are not enough mental health specialists. THP is part of the WHO’s flagship mental health gap action programme (mhGAP) and has been translated into a number of languages and is being implemented in many countries [20, 23–25].

Based on the current evidence, there is reason to believe that THP would be an effective and feasible way of managing perinatal depression in China. The aim of this study was to translate and adapt THP for the Chinese population and to establish its acceptability, comprehensibility and cultural relevance, when delivered to depressed women in perinatal period by non-specialists.

## Methods

### Procedures

This study was conducted in two phases from January to July 2019.

#### Phase 1: translation and adaptation

For this phase, the translation and adaptation of the THP manual as well as handbook and health calendar was done. English language versions of programme materials including the original THP handbook and health calendar were provided by Human Development Research Foundation where the original THP was developed. Translation and adaptation was conducted in three steps: a) Translation by a team of bilingual researchers; b) adaptation by an expert group, and; c) pre-testing and cognitive interviewing. First, translation and adaptation of THP intervention materials (manual, handbook, health calendar) was done according to Bernal framework of translation and adaptation of interventions. The Framework not only provides a useful method of documentation but also allows the translators and expert group to focus on the key dimensions that need to be adapted [26]. Pre-testing was done using cognitive interviewing to guide the adaptation processes. Cognitive interviewing is a commonly used method to check the accuracy of health questionnaires or interventions developed in one cultural context and then translated for use in a different language and culture [27–29].

##### Translation

This was carried out by a team of three researchers (XL, JY, NY), led by a health professional (XL), who was trained in THP. She was knowledgeable of the English language and culture, with Mandarin as her mother tongue.

##### Adaptation by expert group

An expert group was established***,*** which included the author of the original intervention manual (AR), a master trainer (AN), a bilingual health expert (XL), potential user (i.e. Mothers), and a delivery agent (i.e., THP trained nurses). The translation team discussed any problematic items with the group during translation. Through frequent discussions with the expert group, equivalence was achieved at both the technical level (i.e., grammar, tense, acceptable level of abstraction) as well as the conceptual level (obtaining an identical meaning of concepts, which may have different cultural expression, e.g., idioms or metaphors). The expert group was also responsible for revising the cultural adaptation of the language and pictures, and for advising about the context and conditions for the delivery of the programme. The group supervised a Chinese artist to re-draw pictures to depict Chinese women, families and settings.

##### Pre-testing and cognitive interviewing

Following the translation and adaptation processes, an adapted ‘cognitive interviewing’ approach was used to validate the Chinese version THP. Trained health professionals facilitated cognitive interviews of 8 pregnant mothers. During these interviews, the interviewer narrated brief segments of the adapted intervention materials, and images from the illustrated manual were shown to the participants, followed by questions focused on acceptability, comprehensibility and cultural relevance of the adapted intervention.

##### Documentation

Throughout the translation and adaptation process, proposed session-wise and module-wise changes were noted by the translation team using a structured form that included the eight adaptation dimensions described by Bernal framework. Examples of documentation of some key adaptations can be seen in Table 1.

#### Phase 2: field-testing

Following completion of the translation and adaptation process, five THP trained nurses delivered seven face-to-face sessions of adapted version of THP to 15 perinatally depressed women. Post intervention delivery, a programme survey with fixed choice open-ended questions was conducted with the mothers, their family members and the nurses who delivered the intervention.

This was not a pilot study but a field-test of the intervention. While the two terms are often used interchangeably, field-testing does not involve the collection of data to provide an quantitative analysis of the validity or effectiveness of an instrument or intervention, rather it is meant to seek opinions and feedback from a variety of users, usually to determine the appropriateness of a concept in a particular context [30, 31].

##### Settings and participants

The study was undertaken in Yanta district of Xi’an, Shaanxi, in Northwestern China. As the most populous city in Northwest China, Xi’an has a population of 9 million, with 67% urban and 32% rural population. In 2017 with the birth rate of 12.62‰, there were over 100,000 newborns in Xi’an. There are 3.2 doctors and 4.4 nurses per 1000 residents. Hospital delivery rate in Xi’an is 99.9% [32]. However, there are very few mental health specialists [33]. Considering almost all women, from every socioeconomic strata, have access to antenatal and postnatal care, we aimed to adapt THP so it could be delivered by nurses providing perinatal services to women. Our targeted delivery agents were thus nurses working in perinatal natal care settings.

For field-testing, we targeted a selected sample of mothers who received the intervention, their family members and nurses who delivered the intervention. 15 Chinese women of 18 or above years, who were at least 25 weeks pregnant, and scored 5 or above on the Patient Health Questionnaire-9 (PHQ-9) [34] were purposively selected to participate (Table 2). Women who met the eligibility criteria were informed of the study and after an informed consent THP materials including THP handbook and health calendar were given to the participants. Five local nurses, who were trained by THP specialist, delivered face-to-face THP sessions to the 15 mothers.

Post-intervention, all participants including 15 mothers, 5 family members of the mothers, and 5 nurses were administered semi-structured questionnaires, which included study-specific, fixed choice and open ended questions to assess acceptability, comprehensibility and cultural relevance of the adapted intervention. Participants were encouraged to be open about their response.

The purpose of the field-test was to obtain feedback about the acceptability, comprehensibility and cultural relevance of the adapted version of THP provided by the perinatal women who received the intervention, their family members and the nurses who delivered the intervention. The participants were purposively selected to represent a cross-section of the population that would be the ultimate consumers of the intervention. As the purpose of the study was not to test any specific hypotheses about validity or effectiveness, power calculations were not conducted.

## Results

### Translation and adaptation process

**Fig. 1:**
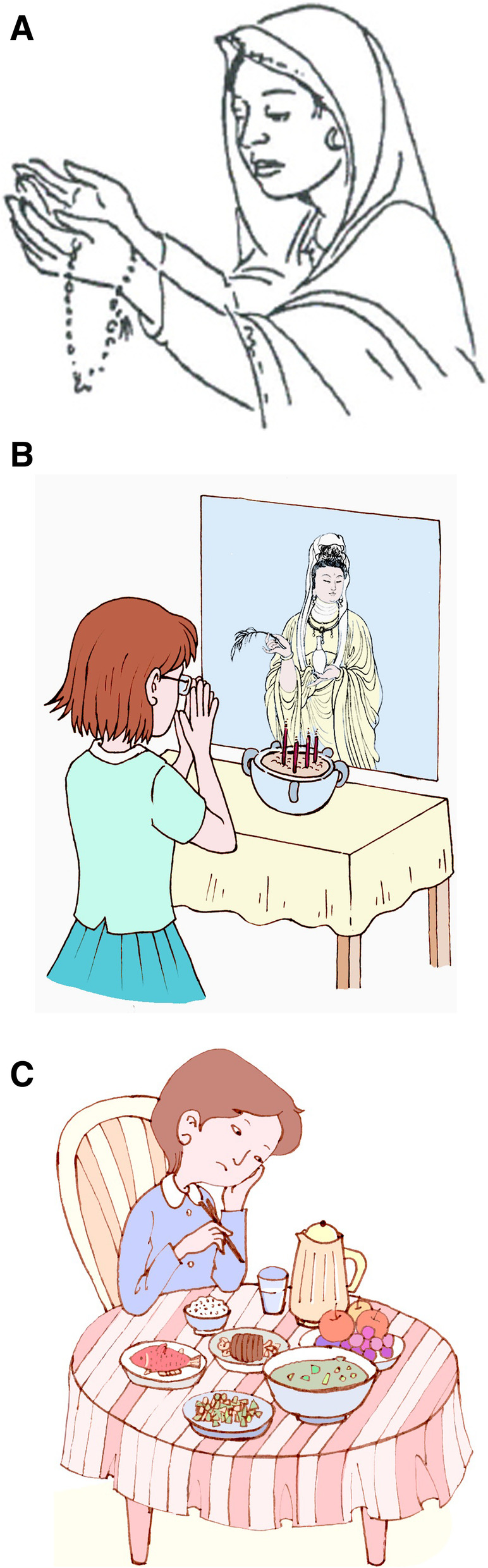
*(Picture A is an image of a women praying in the original context of THP in Pakistani settings and Picture B is the adapted image of a women praying in China)*

It was found that the core structure, techniques and elements of the intervention were culturally compatible and did not require major changes (Fig. 1A, B). However, subtle but critical adaptations were required in all of the domains. Key areas and examples of adaptations are given in Table 1. In line with Bernal’s framework, the translator aimed at translating the conceptual equivalent of a phrase, rather than a word-for-word translation, keeping their target audience in mind. (i) The *language* was kept simple, clear and concise by avoiding the long and complex sentences. Specialized terms and jargon were avoided. The language was kept such that the mothers could relate to its content and therefore develop a supportive relationship with the lay therapist. To achieve this, a number of culturally relevant (ii) *Metaphors* were employed. For example, the three steps of the Thinking Healthy approach were conveyed through the story of a typical Chinese mother. This proved helpful in conveying the key messages. (iii) *The content* of the intervention material was adapted so it referred to local customs and practice. For example, local methods of meditation and prayer were incorporated.

**Table 1:** Bernal framework of adaptations and examples of key adaptations

Dimension	Operationalization	Examples of key adaptations*
**1. Language**	Emotional expression, gestures, verbal style	• All the materials were translated into the simplified Mandarin. • Language was kept specifically colloquial rather than formal. Translations were conceptual rather than literal and word-to-word, to make the participants understand the underlying ideas and the concepts of THP
**2. Metaphors**	Symbols and concepts; sayings / proverbs	• Addition of common Chinese proverb *“The old man is a treasure”* to elaborate the importance of the mother’s relationship with the elderly family members around her as per Chinese family culture. • Addition of local idioms like *“How do you know the pleasure of fish?”* – which implies that do not judge the situations based only on your thoughts. • Images embodying local avatar were used (Fig. 1C). • A made-up story of a Chinese mother “*Huifang*” experiencing perinatal depression and having the life circumstances closer to the general population of Xi’an was added. The purpose was to help the participants understand the occurrence and preventive measures of perinatal depression as clearly as possible.
**3. Content**	Familiarity with local values, customs and traditions	• Local traditions and practices of treatment (e.g. meditation, prayers, acupoint massage) were referred. • Somatic concepts (Chinese traditional model of body, health and sub-health) were added. • Examples of the stressors that were culturally and socially suitable, e.g. Family conflicts in the relationship of mother-in-law and daughter-in-law; occupational stresses during pregnancy were added.
**4. Concepts**	Constructs of theoretical model - how clients problem is perceived and communicated, including availability of locally used terms for theoretical concepts	• Family concept (e.g., single child with multiple carers) was adapted. • Social concepts (e.g., more egalitarian status of women) were adapted. There was a challenging situation in the original manual where the mothers are not allowed by the family to take part in the program. This was replaced with the situation where the mothers are not willing to take part in the programme. • South Asian concept of the ‘evil eye’ was substituted with the similar but slightly different concept of ‘the curse’. • Cultural concepts (i.e. suffering is caused by a ‘curse’; and one month of the mother’s rest and confinement period after delivering the baby were added in the content.
**5. Goals**	Reflecting knowledge of values, culture, customs and traditions	• To encourage active participation of the mothers, additional activities about problem solving discussions were added and mothers were given more chance of active-participation by choosing activities from health calendar rather than the therapist being prescriptive. • Healthy activities that were locally relevant (e.g., tai chi, gong qi and exercises) were added.
**6.Healthcare context**	Health systems within which intervention is delivered	• All the THP materials were modified for integration into the Chinese perinatal healthcare context.
**7. People**	Delivery agent and the client-counselor relationship	• Nurses were identified as acceptable delivery agent as they were seen as custodians of pregnancy and postpartum care.
**8. Methods**	Procedures to deliver intervention	As majority of Chinese women go to work and cannot spare time for THP sessions separately, the frequency THP face-to-face individual sessions was reduced from 16 home-based sessions to 7 sessions to be delivered to the mother at the hospital. So that number of sessions remains congruent with the number of women’s usual anticipated perinatal checkup visits at the hospital to ensure the sustainable delivery of THP sessions.

Similarly, the content was modified to use local expressions of distress and body symptoms and referred to the traditional Chinese concepts. Besides the content delivered by the nurses, the health calendars, which is related to behavioral activation of mothers, is also adapted for the sociocultural milieu. (iv) The *Concepts* of family, society and culture were examined carefully and adapted. In China the family is considered to be a central institution. For instance the role of family was modified to account for grandparents as family caregivers of a single-child family. The details about egalitarian social status of women in China were also reflected in the adapted text. (v) *Goals –* keeping cultural and traditional differences in view, more emphasis was kept on client derived goals rather than prescriptive. The goals of the therapy, such as the ‘homework activities’ related to behavioral activation and tasks set for the mother between sessions also needed adaptation to for the sociocultural milieu. (vi) The *Context* - Adaptations to the context were important to ensure that the intervention could be delivered feasibly with the healthcare systems. Some THP sessions were abbreviated to match the frequency of routine visits of women for perinatal care 7–10 visits. (vii) *People -*Nurses providing perinatal services were perceived to be perfectly acceptable as delivery agents given their role in care of the perinatal women. (viii) *Methods* - All the pictures were redrawn to match the Chinese settings and culture and the health calendar activities were made compatible with the healthcare system.

### Field-testing

#### Participant characteristics

The socio-demographic characteristics of the participant mothers were similar to the local population of Xi’an, regarding social and demographic status and family structure. Participating women were young, educated and workingwomen without any accidental severe life events. All of the mothers received complete intervention of 7 adapted sessions. Details of socio-demographic details of the 15 recipient women, their family members and THP facilitator nurses are given in Table 2. All participants completed the post intervention evaluation. The feedback from the field-test is summarized in.

**Table 2:** Socio-demographic characteristics of the participants

Socio-demographic characteristics	Mothers (***n*** = 15)	Family members (***n*** = 5)	THP facilitator Nurses (n = 5)
**Mean age [range]**	27 [22–32]	49 [35–63]	32 [27–35]
**Occupation**			
Employed	6	3	5
Student	3	–	0
House wife	6	2	–
**Education level**			
Graduation	10	3	5
High School	3	–	–
Secondary school	2	–	–
None	–	2	–
**Marital status**			
Married	15	5	5
**Family structure**			
Nuclear	9	2	3
Joint	6	3	2
**Parity (n)**			
Nulliparous	11	N/A	N/A
Multiparous	4	N/A	N/A
**Household members, median [range]**	3 [4–6]	3 [4–6]	N/A
**Median gestation weeks [range]**	25 [22–28]	N/A	N/A
**Coincidental adverse events**	–	–	N/A
**Ever experienced fear of husband over the past year**			
Never	12	N/A	N/A
Sometimes or often	3	N/A	N/A

#### Feedback from participants

*Acceptability*: A majority of the participants found THP sessions appropriate and meaningful. Ten out of 15 mothers found THP sessions acceptable and enjoyable. *“I can see how small changes in my routine can help my health and my child’s wellbeing. It’s very convenient to follow, I think all the pregnant women and mothers can use this.”*- a mother’s words. Nurses were able to see how mothers were able to engage in the discussions after a couple of THP sessions. All the nurses found the content acceptable and deliverable, *“While I was delivering the programme to the mother, I saw how she begin to bond with me befriended me and it made my work easier”* – quote from a nurse. Four out of five of the family members of the mothers found THP helpful and acceptable while only one rendered it somewhat helpful (Table 3).

**Table 3:** Participant’s response to fixed-choice questions about THP sessions

Themes	Mothers (n = 15)	Family members (n = 5)	THP Facilitator Nurses (n = 5)
**Perceived helpfulness of THP content**			
Very helpful	10	4	3
Helpful	4	1	2
Somewhat helpful	1	–	–
Not helpful	–	–	–
**Perceived Comprehensibility of THP content**			
Easy to understand	11	3	4
Understandable	3	1	1
Somewhat understandable	2	1	–
Difficult to understand	–	–	–
**View about THP illustrations**			
Helpful in understanding concepts	12	3	3
Needs improvement	3	2	2
Not conveying anything	–	–	–
**Impact of THP homework activities**			
Extremely useful	9	4	3
Useful	4	1	2
A little useful	2	–	–
Not useful at all	–	–	–
**Cultural relevance of THP content**			
Suitable in Chinese culture	14	4	5
Needs improvement	1	1	–
Not suitable at all	–	–	–
**Suitable delivery agent for the programme**			
Nurses	10	2	4
Specialized Psychiatrists	3	2	1
Others	2	1	0

##### Comprehensibility

All the participants found the translated version of THP clear and understandable. Eleven out of 15mothers, three out of five family members, and four out of five nurses found the intervention content and concepts easy to understand. A mother stated, *“All the messages are very simple and clear, pictures are self explanatory. You can learn very easily”. Nurses* found that mother were able to identify the unhealthy thoughts and follow the THP sessions more clearly as the sessions go by *“In beginning she (mother) were not interacting much but with the passage of time it became easy for me to work with her”-* a nurse’s remarks. Participants were able to understand the underlying concept of identifying unhealthy thoughts, replacing them with healthy thoughts and practicing health activities. Family members of the mothers also found the language and the concept of the THP sessions understandable.

##### Cultural relevance

Fourteen out of 15 mothers found the content and the delivery of THP sessions culturally relevant. Almost all participants found the intervention and the illustrations culturally appropriate. Programme materials (such as mood chart and health calendar) were found to be relevant and helpful by the majority of the mothers and their family members. *“The story of Huifang is very good, we can easily understand and relate to it, pictures are very good- I like these, they can help in better understanding of situations” –* a mother’s comments. The healthy activities suggested between sessions (homework) were found very useful or useful by most of the participants. *“The health calendar helped me a lot to monitor the involvement of the mother in the programme, these are healthy and simple daily life activities for her wellbeing”.*

*Preferred delivery agents of THP*: Importantly, the majority of (16 out of 25) participants found nurses to be the suitable delivery agents, only a very small (6 out of 25) minority that would prefer a specialist mental health professional. A mother, when asked about her opinion in this regard stated, *“Nurses are better, we can talk to them easily and trust their guidance. My nurse (who delivered THP) was just like my friend”*. THP trained Nurses were able to deliver the intervention well, *“I had no problem in delivering it, it’s more like befriending the mother and guiding them in their daily life”*– a nurse’s views. Results of the post-intervention survey are summarized in Table 3.

## Discussion

Perinatal mental health is a global public health priority. Evidence-based psychosocial interventions such as the Thinking Healthy Programme, recommended as first-line treatment by the WHO, need to be rapidly adapted to the cultural and health-care context of populations before implementation. Appropriate translation and adaptation of intervention materials is a vital first step towards evaluation and implementation of any intervention. This study provides a systematic way of translating and adapting an intervention developed in one culture for use in another very different language and culture. It therefore provides a replicable model that other researchers can us not only to translate, but to also document the changes made to the original intervention.

The study shows that the core structure, process and techniques of the Thinking Healthy Programme were culturally compatible with our target Chinese population and did not require major changes. However, subtle but critical adaptations were required which were done relatively quickly using standard methods involving participant consensus and rapid field-testing. These subtle conceptual changes were critical to make the intervention acceptable. The study thus provides a methodology that can be considered for rapid translation and adaptation as well as a prototype adapted manual that can be used for further implementation and evaluation studies in the Chinese context.

The original Thinking Healthy Programme manual recommended local adaptations to be conducted prior to implementation, but did not provide a standardized methodology for conducting such adaptations [19]. Thus, researchers have employed different methods. The utilization of Bernal framework facilitated structured content adaptations and documentation across various domains, allowing other researchers to clearly see the level of changes made to the original version and their rationale. Cognitive interviewing was found to be an effective and rapid method to pre-test the adapted version with the target population prior to field-testing. This method, recommended by the WHO has been used successfully to rapidly adapt a self-help intervention for psychological distress among refugees in an African context [29]. In Peru, the Replicating Effective Programmes (REP) framework was used to guide the implementation process of THP, but the authors relied on a pre-translated Spanish version of the manual conducted by a community-based organisation for their local programmes [24, 35]. It is therefore difficult to evaluate the process of adaptation and the key changes made to the original THP and their rationale. In Vietnam, an adaptation framework derived from the WHO’s International Management of Childhood Illnesses adaptation toolkit was used to translate and adapt the intervention [36]. As in our study, it also involved participant consensus and field testing [25]. THP-Vietnam findings were similar to our study, i.e., THP core structure, processes and techniques were transferrable across cultures but required some linguistic and cultural adaptations. The results of our study, and studies conducted in other settings indicate that the key constructs of the Thinking Healthy Programme are culturally transferable [22, 24, 25].

Furthermore, the intervention is perceived to be acceptable by the target population. THP is based on principles of cognitive behavior therapy (CBT). This is corroborated by several small-scale studies conducted in various regions of China, indicating that similar CBT based interventions were effective in the local populations [37–39]. However, the main advantage of the Thinking Healthy Programme is that it is designed to be integrated into existing health systems and therefore scalable. Our adapted version of THP incorporates local concepts (such as ‘Confinement month’, traditional exercises (such as Tai Chi and Qi gong) and traditional practices (such as acupoint massage). There is some evidence (although of poor quality studies with small sample sizes) that these traditional practices and exercise are very effective when combined with conventional therapy [40].

THP can be a potential value added component to the China’s existing universal healthcare programme for the health and wellbeing of the pregnant women, the mothers and the infants. China is making efforts towards integration of hospital and community settings for mental health equity. In 2004, Project 686 attempted to integrate the joint resources of psychiatry hospitals and community health systems [33]. Key innovation of project 686 was to Integrate of management and treatment for severe mental disorders in hospital and community settings. Under this project trainings were provided for psychiatrists, clinical nurses, administrators, community health workers, policemen and patients’ families in order to provide a systematic care for those in need. From 2004 to 2015, they successfully trained 11,457 psychiatrists, 6600 trainees, 385,700 patients’ family members [41]. Another similar integration of hospital and community settings can be proposed for the treatment of perinatal depression in Chinese women. China has a strong three tier primary health care model with community level health centers staffed by trained health nurses accessible to all. The community health stations are very organized national organization, established throughout the country at all social levels, including small communities [42]. Their purpose is to work towards health and wellbeing of community residents. This suggests that primary health care is an ideal site for the integration of mental health intervention like THP into maternal health promotion programmes, with the hospital-based specialist workforce taking the responsibility of training and supervision of the intervention. However, in order to achieve effective coverage and successful integration of mental health services into existing health care model, both supply-side barriers and demand-side barriers related to stigma and range of mental disorders need to be addressed. Investing in increasing demand for mental health services through active engagement of the community, to strengthen service user leadership can be a potential way forward. Task sharing with community-based workers in a collaborative stepped-care model is a feasible approach for scale up and integration within the national health care systems [43]. Interventions such as the Thinking Healthy Programme ensure this possible, and the approach also ensures that the content and delivery of mental health interventions are culturally and contextually appropriate.

### Limitations

Due to narrow scope of the study and resource constraints, impact of the adapted intervention on the mental health of the mothers was not analyzed. Field-testing did not involve detailed process evaluation and was kept brief with small group of participants not recruited systematically. While the survey conducted was anonymous, still there may have been subject bias, the views expressed by women participants, delivery agents and community representatives appeared to follow a similar pattern. We stress that our study does not demonstrate the effectiveness of the intervention in treating perinatal depression – this would be done using methods such as randomized controlled trials. We plan to conduct this as a next step towards establishing this intervention as a candidate for scale-up in the country.

## Conclusion

Following translation and adaptation, preliminary feedback from users and delivery agents indicate that the Thinking Healthy Programme may be transferable to the Chinese cultural and healthcare context. However, further research, including randomized controlled trials, will be required to establish the effectiveness and barriers to scale-up.

## Data Availability

The datasets used and/or analysed during the current study are available from the corresponding author on reasonable request.

## Abbreviations

WHO: World Health Organization
mhGAP: Mental health Gap Action Programme
LMIC: Lower and Middle Income Countries
THP: Thinking Healthy Programme
CBT: Cognitive Behavior Therapy
REP: Replicating Effective Programme

## References

[1] Rahman A, Surkan PJ, Cayetano CE, Rwagatare P, Dickson KE (2013). Grand challenges: integrating maternal mental health into maternal and child health programmes. PLoS Med.

[2] Gaynes BN, Gavin N, Meltzer-Brody S, Lohr KN, Swinson T, Gartlehner G (2005). Perinatal depression: prevalence, screening accuracy, and screening outcomes: summary report: Agency for Healthcare Research and Quality (US).

[3] Austin MP (2004). Antenatal screening and early intervention for “perinatal” distress, depression and anxiety: where to from here?. Arch Womens Ment Health.

[4] Fisher J, Mello MC, Patel V, Rahman A, Tran T, Holton S (2012). Prevalence and determinants of common perinatal mental disorders in women in low-and lower-middle-income countries: a systematic review. Bull World Health Organ.

[5] Grote NK, Bridge JA, Gavin AR, Melville JL, Iyengar S, Katon WJ (2010). A meta-analysis of depression during pregnancy and the risk of preterm birth, low birth weight, and intrauterine growth restriction. Arch Gen Psychiatry.

[6] Evans J, Heron J, Francomb H, Oke S, Golding J (2001). Cohort study of depressed mood during pregnancy and after childbirth. BMJ..

[7] Kurki T, Hiilesmaa V, Raitasalo R, Mattila H, Ylikorkala O (2000). Depression and anxiety in early pregnancy and risk for preeclampsia. Obstet Gynecol.

[8] Lutsiv O, McKinney B, Foster G, Taylor VH, Pullenayegum E, McDonald SD (2015). Pregnancy complications associated with the co-prevalence of excess maternal weight and depression. Int J Obes.

[9] Bitew T, Hanlon C, Kebede E, Honikman S, Fekadu A (2017). Antenatal depressive symptoms and perinatal complications: a prospective study in rural Ethiopia. BMC Psychiatry.

[10] Bernard-Bonnin A-C, Canadian Paediatric S, Mental H, Developmental DC (2004). Maternal depression and child development. Paediatr Child Health.

[11] Rahman A, Bunn J, Lovel H, Creed F (2007). Association between antenatal depression and low birthweight in a developing country. Acta Psychiatr Scand.

[12] Bergman K, Sarkar P, O'Connor TG, Modi N, Glover V (2007). Maternal stress during pregnancy predicts cognitive ability and fearfulness in infancy. J Am Acad Child Adolesc Psychiatry.

[13] Beijers R, Jansen J, Riksen-Walraven M, de Weerth C (2010). Maternal prenatal anxiety and stress predict infant illnesses and health complaints. Pediatrics..

[14] Mu T-Y, Li Y-H, Pan H-F, Zhang L, Zha D-H, Zhang C-L (2019). Postpartum depressive mood (PDM) among Chinese women: a meta-analysis. Archives of women's mental health.

[15] National Bureau of Statistics of China. Statistical Yearbook of China's Health and Family Planning, 2018.http://www.stats.gov.cn/tjsj/ndsj/2018/indexeh.htm. Accessed 18 Dec 2019.

[16] Patel V, Xiao S, Chen H, Hanna F, Jotheeswaran AT, Luo D (2016). The magnitude of and health system responses to the mental health treatment gap in adults in India and China. Lancet.

[17] Guo Y, Huang Y (2019). Realising equity in maternal health: China's successes and challenges. Lancet.

[18] Sockol LE, Epperson CN, Barber JP (2011). A meta-analysis of treatments for perinatal depression. Clin Psychol Rev.

[19] Rahman A, Fisher J, Bower P, Luchters S, Tran T, Yasamy MT (2013). Interventions for common perinatal mental disorders in women in low-and middle-income countries: a systematic review and meta-analysis. Bull World Health Organ.

[20] World Health Organization. Thinking Healthy - A manual for psychological management of perinatal depression.2015.https://www.who.int/mental_health/maternal-child/thinking_healthy. Accessed 15 Dec 2019.

[21] Rahman A, Malik A, Sikander S, Roberts C, Creed F (2008). Cognitive behaviour therapy-based intervention by community health workers for mothers with depression and their infants in rural Pakistan: a cluster-randomised controlled trial. Lancet.

[22] Fuhr DC, Weobong B, Lazarus A, Vanobberghen F, Weiss HA, Singla DR (2019). Delivering the thinking healthy Programme for perinatal depression through peers: an individually randomised controlled trial in India. Lancet Psychiatry.

[23] Sikander S, Ahmad I, Atif N, Zaidi A, Vanobberghen F, Weiss HA (2019). Delivering the thinking healthy Programme for perinatal depression through volunteer peers: a cluster randomised controlled trial in Pakistan. Lancet Psychiatry.

[24] Eappen BS, Aguilar M, Ramos K, Contreras C, Prom MC, Scorza P, et al. Preparing to launch the ‘thinking healthy Programme’perinatal depression intervention in urban Lima, Peru: experiences from the field. Global Mental Health. 2018;5. 10.1017/gmh.2018.32 PMC6315282 30637114

[25] Fisher J, Nguyen H, Mannava P, Tran H, Dam T, Tran H (2014). Translation, cultural adaptation and field-testing of the thinking healthy program for Vietnam. Glob Health.

[26] Bernal G, Jiménez-Chafey MI, Rodriguez MMD (2009). Cultural adaptation of treatments: a resource for considering culture in evidence-based practice. Professional Psychology Research and Practice.

[27] Beatty PC, Willis GB (2007). Research synthesis: the practice of cognitive interviewing. Public Opin Q.

[28] Carbone ET, Campbell MK, Honess-Morreale L (2002). Use of cognitive interview techniques in the development of nutrition surveys and interactive nutrition messages for low-income populations. J Am Diet Assoc.

[29] Tol WA, Augustinavicius J, Carswell K, Leku MR, Adaku A, Brown FL (2018). Feasibility of a guided self-help intervention to reduce psychological distress in south Sudanese refugee women in Uganda. World Psychiatry.

[30] Carter PD (2002). Building purposeful action: action methods and action research. Educational Action Research.

[31] Howie P, Bagnall R (2017). A methodology for field-testing concepts through expert practitioner engagement. Int J Soc Res Methodol.

[32] China Statistical Press. Xi'an Statistical Yearbook. 2017. https://www.chinayearbooks.com/xian-statistical-yearbook-2017.html. Accessed 2 Dec 2019.

[33] Xiang Y-T, Yu X, Sartorius N, Ungvari GS, Chiu HFK (2012). Mental health in China: challenges and progress. Lancet.

[34] Wang W, Bian Q, Zhao Y, Li X, Wang W, Du J (2014). Reliability and validity of the Chinese version of the patient health questionnaire (PHQ-9) in the general population. Gen Hosp Psychiatry.

[35] Kilbourne AM, Neumann MS, Pincus HA, Bauer MS, Stall R (2007). Implementing evidence-based interventions in health care: application of the replicating effective programs framework. Implement Sci.

[36] World Health Organization. How IMAI (and IMCI) Support National Adaptation And Implementation Of Task Shifting. http://www.who.int/hiv/pub/imai/IMAI_IMCI_taskshifting_brochure.pdf. Accessed 19 Dec 2019.

[37] Ngai F-W, Wong PW-C, Leung K-Y, Chau P-H, Chung K-F (2015). The effect of telephone-based cognitive-behavioral therapy on postnatal depression: a randomized controlled trial. Psychother Psychosom.

[38] Huang J, Li H-J, Wang J, Mao H-J, Jiang W-Y, Zhou H (2015). Prenatal emotion management improves obstetric outcomes: a randomized control study. Int J Clin Exp Med.

[39] Mao HJ, Li HJ, Chiu H, Chan WC, Chen SL (2012). Effectiveness of antenatal emotional self-management training program in prevention of postnatal depression in Chinese women. Perspect Psychiatr Care.

[40] Yin J, Nisar A, Waqas A, Guo Y, Qi W, Wang D, et al. Psychosocial interventions for perinatal depression in China: a systematic review and meta-analysis. J Affect Disord. 2020; (In press). 10.1016/j.jad.2020.03.019 32479331

[41] Ma H (2012). Integration of hospital and community services—the ‘686 project’—is a crucial component in the reform of China's mental health services. Shanghai Arch Psychiatry.

[42] Pan X, Dib HH, Wang X, Zhang H (2006). Service utilization in community health centers in China: a comparison analysis with local hospitals. BMC Health Serv Res.

[43] Liu S, Page A (2016). Reforming mental health in China and India. Lancet.

